# Phosphorylation Mimetic of Myosin Regulatory Light Chain Mitigates Cardiomyopathy-Induced Myofilament Impairment in Mouse Models of RCM and DCM

**DOI:** 10.3390/life13071463

**Published:** 2023-06-28

**Authors:** Katarzyna Kazmierczak, Jingsheng Liang, Luis G. Maura, Natissa K. Scott, Danuta Szczesna-Cordary

**Affiliations:** Department of Molecular and Cellular Pharmacology, School of Medicine, University of Miami Miller, Miami, FL 33136, USA; kkazmierczak@med.miami.edu (K.K.); j.liang@med.miami.edu (J.L.); lgm84@miami.edu (L.G.M.); nks71@med.miami.edu (N.K.S.)

**Keywords:** cardiomyopathy, myosin RLC, myosin ELC, N-terminal protein modification, S15D-RLC phosphomimetic, super-relaxed state (SRX), Tg mice

## Abstract

This study focuses on mimicking constitutive phosphorylation in the N-terminus of the myosin regulatory light chain (S15D-RLC) as a rescue strategy for mutation-induced cardiac dysfunction in transgenic (Tg) models of restrictive (RCM) and dilated (DCM) cardiomyopathy caused by mutations in essential (ELC, *MYL3* gene) or regulatory (RLC, *MYL2* gene) light chains of myosin. Phosphomimetic S15D-RLC was reconstituted in left ventricular papillary muscle (LVPM) fibers from two mouse models of cardiomyopathy, RCM-E143K ELC and DCM-D94A RLC, along with their corresponding Tg-ELC and Tg-RLC wild-type (WT) mice. The beneficial effects of S15D-RLC in rescuing cardiac function were manifested by the S15D-RLC-induced destabilization of the super-relaxed (SRX) state that was observed in both models of cardiomyopathy. S15D-RLC promoted a shift from the SRX state to the disordered relaxed (DRX) state, increasing the number of heads readily available to interact with actin and produce force. Additionally, S15D-RLC reconstituted with fibers demonstrated significantly higher maximal isometric force per cross-section of muscle compared with reconstitution with WT-RLC protein. The effects of the phosphomimetic S15D-RLC were compared with those observed for Omecamtiv Mecarbil (OM), a myosin activator shown to bind to the catalytic site of cardiac myosin and increase myocardial contractility. A similar SRX↔DRX equilibrium shift was observed in OM-treated fibers as in S15D-RLC-reconstituted preparations. Additionally, treatment with OM resulted in significantly higher maximal pCa 4 force per cross-section of muscle fibers in both cardiomyopathy models. Our results suggest that both treatments with S15D-RLC and OM may improve the function of myosin motors and cardiac muscle contraction in RCM-ELC and DCM-RLC mice.

## 1. Introduction

Cardiac myosin powers heart contraction through the ATP-dependent cyclic interactions between myosin cross-bridges and actin-based thin filaments comprised of a tropomyosin (Tm) and troponin (Tn) complex [1,2]. In addition to the known Ca^2+^-Tm-Tn regulatory system [3], myosin, together with the regulatory (RLC) and essential (ELC) light chains, plays an important role in the regulation of muscle contractility and heart performance [4,5]. Many genetic mutations in cardiac sarcomeric proteins, including myosin RLC and ELC, were found to be responsible for the modulation of myosin motor function and affecting heart performance [6,7,8,9,10]. As myosin light chains are important regulators of the actin–myosin interaction, they constitute a potential drug target for rescue strategies to ameliorate or reverse myosin motor dysfunction and abnormal disease phenotypes [11,12,13,14]. 

One such strategy is associated with the myosin light-chain kinase (MLCK)-dependent phosphorylation of cardiac RLC, which has been shown in many studies to be critical for normal heart function [15,16,17]. Myosin regulatory light-chain phosphorylation is a crucial determinant of myosin motor function and heart performance in healthy and cardiomyopathic hearts; still, little is known about the underlying mechanisms. Cardiac MLCK, the enzyme that phosphorylates RLC, has been shown to play a role in cardiogenesis [18] and myofibrillogenesis [19]. The upregulation of MLCK is considered a mechanism to promote sarcomere reassembly and improve contractility in a failing heart [18]. Constitutive RLC phosphorylation at ~40% has been shown to stabilize myosin motor function in continuously beating hearts [20]. In the human heart, myosin RLC phosphorylation occurs at serine 15 (Ser-15), and this site was recently shown to be superior to other potential phosphorylation sites of RLC [21]. We also showed that the phosphomimetic RLC variant, in which Ser-15 is replaced by aspartic acid (S15D), mitigates detrimental hypertrophic cardiomyopathy (HCM) phenotypes in vivo [22] and in vitro [21] when placed in the background of HCM-associated RLC mutations. In the current study, we aimed to test whether the adverse effects of restrictive cardiomyopathy (RCM OMIM 115210)-E143K ELC and dilated cardiomyopathy (DCM OMIM 606685)-D94A RLC mutations that exert severe myofilament impairments could be rescued by phosphomimetic S15D-RLC reconstituted in Tg-E143K versus Tg-WT ELC fibers and Tg-D94A versus Tg-WT RLC preparations. 

RCM is characterized by enlarged atria, stiffness of normal-sized ventricles, and impaired ventricular filling leading to diastolic dysfunction [23]. The E143K ELC mutation was first identified in a young male patient during a medical evaluation due to the premature death of his siblings [24]. An incidence of death at seven years of age was reported in a distant family member [24]. In another report, E143K was discovered in a 22-year-old female patient diagnosed with RCM and heart failure (HF) while awaiting heart transplantation [25]. The RCM-E143K animal model used in the current study faithfully recapitulated major RCM features, e.g., cardiac dysfunction, stiff ventricles, and physiological, morphologic, and metabolic remodeling consistent with the RCM phenotype [26]. 

On the other hand, DCM is a progressive heart disease characterized by left ventricular dilation and severely reduced contractile function, and it is one of the leading causes of HF in humans [27]. Huang et al. described the first sarcomeric protein mutation in the *MYL2* gene (RLC-D94A) associated with DCM [9]. The D94A mutation was discovered in a registered DCM family using exome sequencing, rather than an established DCM gene panel [9]. Interestingly, Tg-D94A mice, the source of the cardiac muscle preparations used in the current report, have shown that hypo-contractile myosin motors trigger aberrant sarcomeric remodeling in mutant hearts, leading to the development of DCM [28]. 

Tg-E143K and Tg-D94A models of RCM and DCM, respectively, have been instrumental in confirming the perception that the mutations identified in *MYL3* and *MYL2* genes cause cardiomyopathy with typical features of human disease observed in these mouse models. Left ventricular papillary muscle (LVPM) fibers from Tg mice were depleted of endogenous RLC and reconstituted with bacterially expressed human ventricular S15D-RLC protein. The data showed beneficial effects of phosphomimetic RLC on the myosin contractile and energetic states in the RCM and DCM myocardium and comparable to treatment with Omecamtiv Mecarbil (OM), a myosin activator developed to increase heart contractility in acute HF and treat systolic dysfunction [29,30].

## 2. Materials and Methods

### 2.1. Transgenic Mice

All transgenic (Tg) mouse models used in the current study have been characterized in previous papers and used widely in our laboratory [26,28,31]. The following mice were used: Tg-WT RLC (L2, expressing 100% of human ventricular RLC, Swiss-Prot: P10916), Tg-D94A RLC (L1 and L2 expressing 53% and 50% human ventricular mutant D94A RLC), and Tg-WT ELC (L1 and L4, expressing 76% and 71% of human ventricular ELC, Swiss-Prot: P08590), and one Tg-E143K ELC (L2 expressing 55% human ventricular mutant E143K ELC). Male (M) and female (F) mice were used in the experiments.

### 2.2. Recombinant RLC Proteins—Cloning, Expression, and Purification

To generate recombinant RLC proteins, the cDNA of human cardiac WT-RLC, phosphomimetic RLC variant (S15D-RLC), and DCM-D94A RLC mutant were cloned by the reverse transcription–polymerase chain reaction using primers based on the published cDNA RLC sequences (WT RLC—GenBank Accession No. AF020768, S15D RLC—GenBank accession number ON950401) using standard methods as described previously [9,32,33]. The cDNAs for RLC recombinant proteins were constructed with a NcoI site at the N-terminal ATG and a BamHI site following the stop codon to enable ligation into the NcoI-BamHI cloning site of the pET-3d plasmid vector (Novagen, Burlington, MA, USA). DNA sequence determination was performed using the T7 promoter.

The obtained cDNAs were used for transformation into BL21-expression host cells to express proteins in 16 L cultures that were subsequently purified by ion-exchange chromatography using an S-Sepharose column (Sigma-Aldrich, Inc., St. Louis, MO, USA) (equilibrated with 2 M urea, 20 mM sodium citrate, 0.1 mM PMSF, 1 mM DTT, and 0.02% NaN_3_ at pH 6.0), and then a Q-Sepharose column (Sigma-Aldrich, Inc., St. Louis, MO, USA) (equilibrated with 2 M urea, 25 mM Tris–HCl, 0.1 mM PMSF, 1 mM DTT, and 0.02% NaN_3_ at pH 7.5). Recombinant RLC proteins were eluted with 0–450 mM NaCl, and their purity was determined by 15% SDS–PAGE [9,32,33].

### 2.3. Mechanical and ATP Turnover Measurements in Skinned Left Ventricular Papillary Muscle (LVPM) Fibers from Transgenic Tg-RLC and Tg-ELC Mice

#### 2.3.1. Preparation of Skinned LVPM Fibers

LVPM fibers were isolated from the hearts of 3 to 11 mo old Tg mice (Tg-WT RLC, Tg-D94A RLC, Tg-WT ELC, and Tg-E143K ELC). Muscle bundles were separated into small muscle strips (2–3 mm in length and 0.5–1 mm in diameter) in ice-cold pCa 8 solution (10^−8^ M [Ca^2+^], 1 mM free [Mg^2+^] [total MgPr (propionate) = 3.88 mM], 7 mM EGTA, 2.5 mM [Mg-ATP^2−^], 20 mM 3-(n-morpholino) propanesulfonic acid (MOPS) pH 7.0, 15 mM creatine phosphate, and 15 U/mL phosphocreatine kinase with ionic strength = 150 mM adjusted with KPr) that contained 30 mM BDM (Sigma-Aldrich, Inc., St. Louis, MO, USA) and 15% glycerol. They were then placed in pCa 8 solution containing 50% glycerol (storage solution) and incubated for 1 h on ice. Then, the strips were immersed in 1% Triton X-100 (Sigma-Aldrich, Inc., St. Louis, MO, USA), 50/50 (%) pCa 8, and glycerol overnight at 4 °C. The fibers were transferred to a new storage solution and kept at −20 °C until they were used for experiments [28].

#### 2.3.2. Depletion of Endogenous RLC from LVPM and Reconstitution with Recombinant RLC Proteins

Tg mouse LVPM preparations underwent endogenous RLC extraction and reconstitution with recombinant RLC proteins as described previously [21]. Briefly, the extraction of endogenous RLC from ~100 µm wide and ~1.5 mm long strips was performed in the following buffer: 5 mM CDTA (Capitol Scientific, Austin, TX, USA), 1% Triton X-100, 50 mM KCl, 40 mM Tris, 0.6 mM NaN_3_, and 0.2 mM PMSF at pH 8.4, supplemented with 30 µL/10 mL protease inhibitor cocktail (Sigma-Aldrich, Inc., St. Louis, MO, USA) for 40 min at room temperature. During RLC depletion, partial extraction of the endogenous cardiac TnC may occur; therefore, LVPM fibers were incubated with 15 µM TnC (in pCa 8 solution) for 15 min at room temperature. This reaction was followed by the reconstitution of the RLC-depleted muscle strips with the following recombinant proteins: WT-RLC or S15D-RLC for Tg-WT ELC and Tg-E143K ELC, and WT-RLC, S15D-RLC or D94A-RLC for Tg-D94A RLC muscle strips in a pCa 8 solution containing 40 µM of the RLC of interest for 45 min at room temperature. The extraction and reconstitution reactions were carried out with the muscle strips either attached to the arms of a force transducer or free-floating in a 96-well plate. RLC- and TnC-reconstituted LVPM fibers were then washed in pCa 8 buffer and subjected to force–pCa and super-relaxed (SRX) state measurements or transferred to fresh storage solution and kept at −20 °C for 1–5 days until they were used for SRX experiments. The extent of RLC extraction and reconstitution was determined by running experimental LVPM fibers on 15% SDS–PAGE, as described in Kazmierczak et al. [21]. The RLC/ELC band intensities were measured in Tg native, RLC-depleted, and RLC-reconstituted LVPM preparations. The myosin ELC was a loading control, as its content did not change during the RLC depletion/reconstitution procedures [34].

#### 2.3.3. Steady-State Force Development and Ca^2+^-Sensitivity of Force in Reconstituted Muscle Strips

All mechanical experiments were performed on skinned LVPM fibers of approximately ~1.5 mm in length and ~100 μm in diameter. The small muscle strips were attached to the force transducer of the Guth Muscle Research System (Heidelberg, Germany) and then skinned with 1% Triton X-100 in pCa 8 buffer for 30 min (in a 1 mL cuvette). Next, the fibers were washed in pCa 8 buffer (3 times × 5 min) and their sarcomere length was adjusted to 2.1–2.2 μm. To determine the maximal force in the fibers, they were placed in pCa 4 solution, which was the same as the pCa 8 buffer, except the concentration of [Ca^2+^] was 10^−4^ M. Then, LVPM fibers were relaxed in pCa 8 and subjected to force–pCa measurements by immersing them in solutions of increasing Ca^2+^ concentration from pCa 8 to pCa 4. The level of force was measured in each “pCa” solution. Maximal tension readings (in pCa 4) were taken before and after the force–pCa curve, averaged, and then divided by the cross-sectional area of the fibers and expressed in kN/m^2^. The diameter of the fibers was measured at 3 points along the fiber length and averaged. Force–pCa curves for LVPM fibers from Tg-WT ELC, Tg-E143K ELC, Tg-WT RLC, and Tg-D94A RLC were fitted to the Hill equation, yielding the pCa_50_ (free Ca^2+^ concentration producing 50% maximal force) and n_H_ (Hill coefficient and the measure of myofilament cooperativity), as described previously [12,21,28].

#### 2.3.4. Mant-ATP Chase Experiments

LVPM fibers from Tg-WT ELC, Tg-E143K ELC, Tg-WT RLC, and Tg-D94A RLC mice reconstituted with bacterially expressed RLC proteins underwent the SRX studies using ATP turnover measurements, as previously described [13,21,35]. The fluorescent N-methylanthraniloyl (mant)-ATP (Thermo Fisher Scientific, Waltham, MA, USA) was exchanged for nonfluorescent (dark) ATP in skinned LVPM fibers from all groups of mice using IonOptix instrumentation. LVPM fibers were incubated in 250 μM mant-ATP in a rigor solution (120 mM KPr, 5 mM MgPr, 2.5 mM K_2_HPO_4_, 2.5 mM KH_2_PO_4_, 50 mM MOPS, pH 6.8, and fresh 2 mM DTT) until the maximum fluorescence reached a plateau. Then, mant-ATP was chased with 4 mM unlabeled ATP (in rigor buffer), resulting in fluorescence decay, as hydrolyzed mant-ATP was exchanged for dark ATP. Fluorescence intensity isotherms were collected over time and were subsequently fitted to a two-exponential decay equation:

Y = 1 − P1 [1 − exp(−t/T1)] − P2 [1 − exp(−t/T2)](1)

Using a nonlinear least-squares algorithm in GraphPad PRISM version 7 (GraphPad Software, San Diego, CA, USA), the amplitudes of the fast (P1) and slow (P2) phases of fluorescence decay and their respective T1 and T2 lifetimes (in seconds) were derived [12,13,21,28]. P1 was corrected for the fast release of nonspecifically bound mant-ATP in the sample and the correction factor was established using a competition assay, as described in [35,36]. The fraction of nonspecifically bound mant-ATP in LVPM fibers was equal to 0.44 ± 0.02, and the number of myosin heads directly occupying the SRX state was derived as P2/(1 − 0.44) [35].

### 2.4. Treatment of Skinned Muscle Strips with Omecamtiv Mecarbil (OM)

In separate sets of experiments, native LVPM fibers from Tg mice were subjected to treatment with a myosin activator, Omecamtiv Mecarbil (OM) (APExBIO Technology LLC, Houston, TX, USA), versus placebo. All steps/reactions were carried out with the muscle strips either attached to the arms of a force transducer or freely floating in a 96-well plate. Glycerinated LVPM strips were rinsed several times in the pCa 8 solution and then skinned with 1% Triton X-100 dissolved in pCa 8 solution for 30 min, followed by washing in pCa 8 buffer. For fibers undergoing force–pCa measurements, the maximal force (pCa 4) was determined first. Then, the fibers were subjected to treatment with 3 µM OM in pCa 8 buffer or placebo (pCa 8) for 25 min at room temperature. Maximal tension determination and the force–pCa dependence were performed before and after OM treatment, as described in Section 2.3.3. For the ATP turnover experiments, the fibers were incubated with 250 μM mant-ATP in a rigor solution ±3 µM OM for 25 min at room temperature. The mant-ATP chase experiment was performed in the presence and absence of 3 µM OM, as described in Section 2.3.4.

### 2.5. Statistical Analysis

The experimental data showed a normal distribution, and the values are expressed as means ± standard deviation (SD). Statistical significance (*p* < 0.05) was determined using Student’s *t*-test or one-way ANOVA followed by Tukey’s multiple-comparison test (GraphPad PRISM software version 7.0 for Windows). 

## 3. Results

### 3.1. Effect of Phosphomimetic S15D-RLC on Force Measurements in LVPM Fibers from Tg-ELC Mouse Model of RCM

Here, we aimed to investigate the effects of the phosphomimetic S15D-RLC variant on cardiac muscle contraction when reconstituted in skinned LVPM strips from the Tg mouse model of RCM. As we showed recently, the myocardium of Tg-E143K ELC mice is poorly phosphorylated, and this low level of RLC phosphorylation coincides with abnormal myocardial function in the RCM-E143K ELC model [5,26,31]. LVPM fibers from Tg-E143K mice and Tg-WT ELC control were depleted of endogenous RLC and reconstituted with recombinant S15D-RLC or WT-RLC protein. The efficiency of depletion/reconstitution was tested by SDS-PAGE (Figure S1A), and the results are presented in Figure S1B. As published previously [9,13,21,33,34,37,38,39], we could successfully remove endogenous cardiac RLC from LVPM fibers and efficiently reconstitute them with the RLC mutant of choice (Figure S1). 

**Figure 1:**
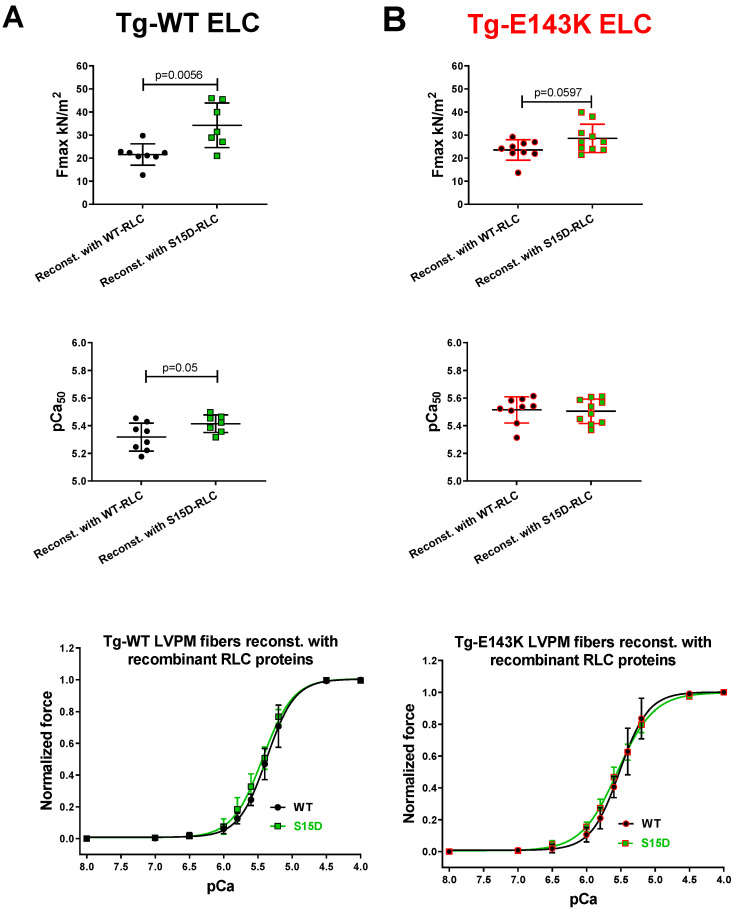
Force measurements in skinned LVPM from Tg-WT ELC (**A**) and Tg-E143K ELC (**B**) mice reconstituted with recombinant RLC proteins. Maximal force (top panels), Ca^2+^-sensitivity of force (middle panels), and force–pCa relationship (bottom panels) were measured in LVPM fibers from Tg-WT ELC and Tg-E143K mice. The fibers were depleted of endogenous RLC and reconstituted with recombinant WT-RLC (black-filled symbols) and S15D-RLC (green-filled symbols) proteins. Data are the average ± SD of n = 7–8 reconstituted LVPM fibers from Tg-WT ELC (2 animals-1F, 1M) and n = 9–10 reconstituted LVPM fibers from Tg-E143K ELC (3 animals-2F, 1M). Significance (*p* values) was calculated by Student’s *t*-test.

The maximal isometric force per cross-section of muscle (in kN/m^2^) and pCa_50_ measured for LVPM from Tg-WT ELC mice reconstituted with recombinant S15D-RLC protein were significantly higher compared with WT-RLC reconstitution (Figure 1A, Table 1). This result is consistent with a previously observed RLC phosphorylation-mediated increase in the Ca^2+^ sensitivity of contraction [15,40]. For LVPM fibers from Tg-E143K mice, Fmax and pCa_50_ were similar between S15D-RLC and WT-RLC reconstitution, but a significant difference between the WT and S15D mutant was observed for the Hill coefficient, n_H_ (Figure 1B, Table 1). The lack of an increase in the calcium sensitivity of force for LVPM fibers from the RCM-E143K mutant reconstituted with S15D-RLC versus WT-RLC was most likely due to the higher Ca^2+^-sensitivity observed in LVPM from Tg-E143K (5.51 ± 0.09) versus Tg-WT ELC (5.32 ± 0.1) reconstituted with WT-RLC, and the addition of phosphomimetic S15D-RLC did not further increase the calcium sensitivity of force in the RCM mutant fibers (Table 1). The lack of a significant increase in Fmax on S15D versus WT RLC reconstitution in Tg-E143K fibers is somewhat puzzling; however, it is hard to predict how pCa 4 force develops in the presence of a disease-causing mutation in response to RLC phosphorylation.

### 3.2. Effect of Phosphomimetic S15D-RLC on the Super-Relaxed State in LVPM Fibers from Tg-ELC and Tg-RLC Mouse Modes of RCM and DCM

Under resting muscle conditions, cardiac myosin can be characterized by two states, a disordered relaxed state (DRX) and a super-relaxed state (SRX) [36]. In DRX, the cross-bridges protrude into the interfilament space, but are restricted from binding to thin filaments. In SRX, an asymmetrical head arrangement along the thick filament axis is formed, called an interacting head motif (IHM), and the cross-bridges cycle with a highly inhibited ATP turnover rate [41,42]. In IHM, the heads interact with each other and the S2 part of the myosin heavy chain (MHC). The IHM is thought to provide a structural basis for the biochemical SRX state [41,43].

#### 3.2.1. Transgenic ELC Mice 

**Figure 2:**
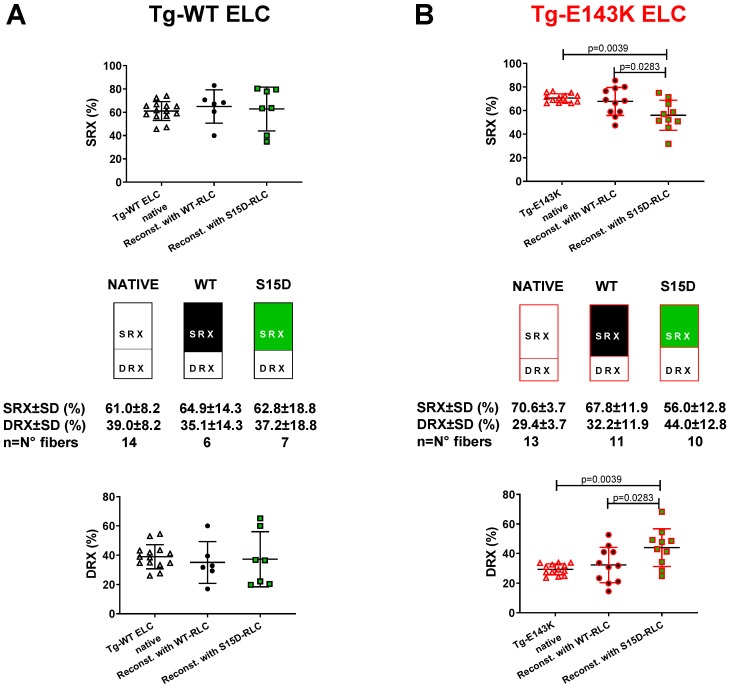
Summary of the SRX study for LVPM fibers from Tg-WT ELC (**A**) and Tg-E143K ELC (**B**) reconstituted with WT-RLC versus phosphomimetic S15D-RLC. The proportion of myosin heads in SRX state (top panels) and DRX states (bottom panels) is shown for native (clear points), WT- (black-filled points), and S15D- (green-filled points) RLC reconstituted Tg fibers. Note that % SRX is increased in native Tg-E143K fibers compared with Tg-WT ELC native controls (~71% versus ~61%). For Tg-E143K fibers reconstituted with WT-RLC or S15D-RLC, reconstitution with the latter significantly decreased % of myosin heads in SRX (~56%) compared with WT-RLC-reconstituted (68%) or native Tg-E143K fibers (71%). Data are expressed as mean ± SD for n = 14 Tg-WT ELC native fibers (4 animals-2F, 2M) and n = 6–7 (4 animals-2F, 2M) for Tg-WT ELC fibers reconstituted with WT-RLC or S15D-RLC. For Tg-E143K set, n = 13 fibers for native Tg-E143K (4 animals-2F, 2M) and n = 10–11 fibers for Tg-E143K (5 animals-3F, 2M) reconstituted with WT-RLC or S15D-RLC were used. The significance was calculated using one-way ANOVA with Tukey’s multiple comparison test.

To study the SRX state and SRX↔DRX equilibrium in reconstituted LVPM fibers from Tg-E143K ELC versus Tg-WT ELC mice, fibers were incubated in a solution containing 250 µM mant-ATP until the fluorescence intensity stabilized [21,35]. Then, upon adding non-labeled ATP (4 mM), fluorescence decay curves versus time were collected and fitted to a two-state exponential equation, as described in Section 2.3.4. No differences were recorded on S15D-RLC versus WT-RLC in Tg-WT ELC fibers showing a 61:39 ratio between SRX and DRX heads (Figure 2A, Table 2). However, LVPM from Tg-E143K mice showed a significant decrease in % SRX heads on S15D-RLC reconstitution, and the SRX/DRX ratio changed from 71:29 (Tg-E143K native) to 56:44 (S15D-RLC), compared with 68:32 for WT-RLC reconstituted (Figure 2B, Table 2). Therefore, the pseudo-phosphorylation of a poorly phosphorylated Tg-E143K model occupying a low-energy SRX state induced a shift in the SRX↔DRX equilibrium toward DRX, making more heads available for muscle contraction compared with WT-RLC reconstituted (Table 2). 

Even though more E143K occupied the DRX state after S15D-RLC reconstitution, they still cycled under relaxation conditions, and whether they switched from the OFF to ON state depended on other contributing factors, e.g., the position of the regulatory proteins on actin/Tm-Tn filaments in Tg-E143K fibers, etc. Therefore, no significant increase in force production was observed in the S15D versus WT-reconstituted Tg-E143K ELC fibers (Figure 1B, Table 1). In conclusion, force production and energy use within the sarcomere is a complex phenomenon, and whether the SRX mechanism thoroughly regulates it is still an open question.

#### 3.2.2. Transgenic RLC Mice

**Figure 3:**
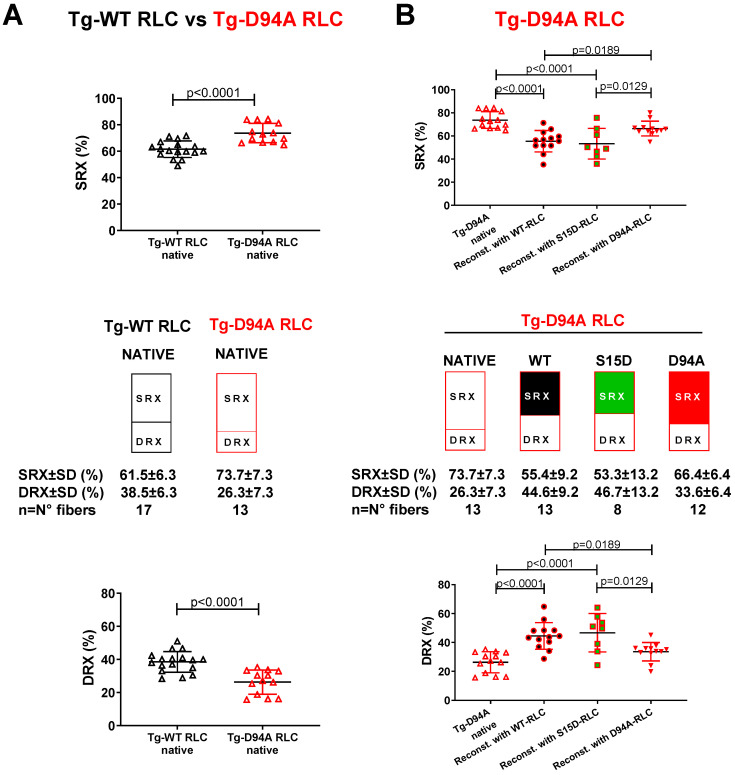
Summary of the SRX study performed on reconstituted LVPM fibers from the model of DCM, Tg-D94A mice. (**A**) SRX comparison between Tg-WT RLC and Tg-D94A RLC mice. (**B**) The proportion of myosin heads in the SRX (top) and DRX (bottom) states. LVPM fibers reconstituted with WT-RLC are shown in black-filled symbols, while those reconstituted with S15D-RLC are shown in green-filled symbols. D94A-RLC-reconstituted fibers are depicted with red-filled symbols. Data are expressed as mean ± SD of n = N° fibers: n = 13, 4 animals-3F, 1M for native Tg-D94A, n = 17, 5 animals-2F, 3M for Tg-WT native, and n = 8–13, 4 animals-1F, 3M for LVPM fibers from Tg-D94A reconstituted with RLC proteins. The significance was calculated by Student’s *t*-test (**A**) and one-way ANOVA with Tukey’s multiple-comparison test (**B**).

We have recently shown the beneficial effect of the phosphomimetic S15D-RLC variant when reconstituted in LVPM fibers preparations from HCM-RLC models of cardiomyopathy [13,21,33]. Here, we aimed to test whether S15D-RLC may rescue some DCM-related abnormalities when reconstituted in LVPM fibers from the DCM RLC model, Tg-D94A mice. In particular, we investigated whether the abnormal SRX/DRX ratio found in this Tg-D94A model previously [35] could return to normal when the endogenous RLC was replaced by recombinant phosphomimetic S15D-RLC protein. LVPM fibers from Tg-D94A mice were depleted of endogenous RLC and reconstituted with recombinant S15D-RLC, and the results were compared with native, WT, or D94A-RLC reconstituted fibers (Figure 3, Table 3). As observed previously [35], LVPM from native Tg-D94A RLC mice demonstrated ~74% myosin heads in the SRX state (Figure 3A), indicating that DCM heads favor the energy-conserving SRX state. Tg-D94A LVPM fibers reconstituted with S15D-RLC protein showed a significant increase in % DRX heads (46.7 ± 13.2) compared with native (26.3 ± 7.3) or D94A (33.6 ± 6.4) reconstituted fibers (Figure 3B, Table 3). The increase in % DRX heads for WT-RLC (44.9 ± 10.2) was similar to S15D-RLC-reconstituted (Table 3). The reason we do not see much difference between WT-RLC and S15D-RLC is that the increase in DRX heads was large, reaching 170% for WT and 178% for S15D-RLC compared with native Tg-D94A DRX heads. These results indicate that RLC does not have to be phosphorylated to work in a rescue manner in this animal model of DCM. Reconstitution with the D94A RLC mutant returned the DCM hypo-contractile phenotype, as seen in native Tg-D94A fibers (Table 3).

### 3.3. Treatment of RCM and DCM Mice with Omecamtiv Mecarbil (OM)

In another series of experiments, we applied Omecamtiv Mecarbil (OM), a positive cardiac inotrope, to skinned LVPM fibers from mice and tested its effect on Ca^2+^-activated muscle contraction and the SRX-to-DRX ratio in resting cardiac muscle. The drug was developed to treat systolic HF by targeting the cardiac MHC to increase myocardial contractility [44]. In clinics, OM has been shown to improve cardiac function in HF patients with a reduced ejection fraction, and among patients who received OM, a lower incidence of HF or death was observed compared with patients receiving a placebo [45]. Research studies on OM showed that the drug could prolong actomyosin attachment and increase the myocardial force by cooperative thin-filament activation [46]. It was also shown that OM’s ability to increase cardiac force production may depend on the phosphorylation of myosin-binding protein C [47].

#### 3.3.1. Steady-State Force and Force–pCa Relationship 

**Figure 4:**
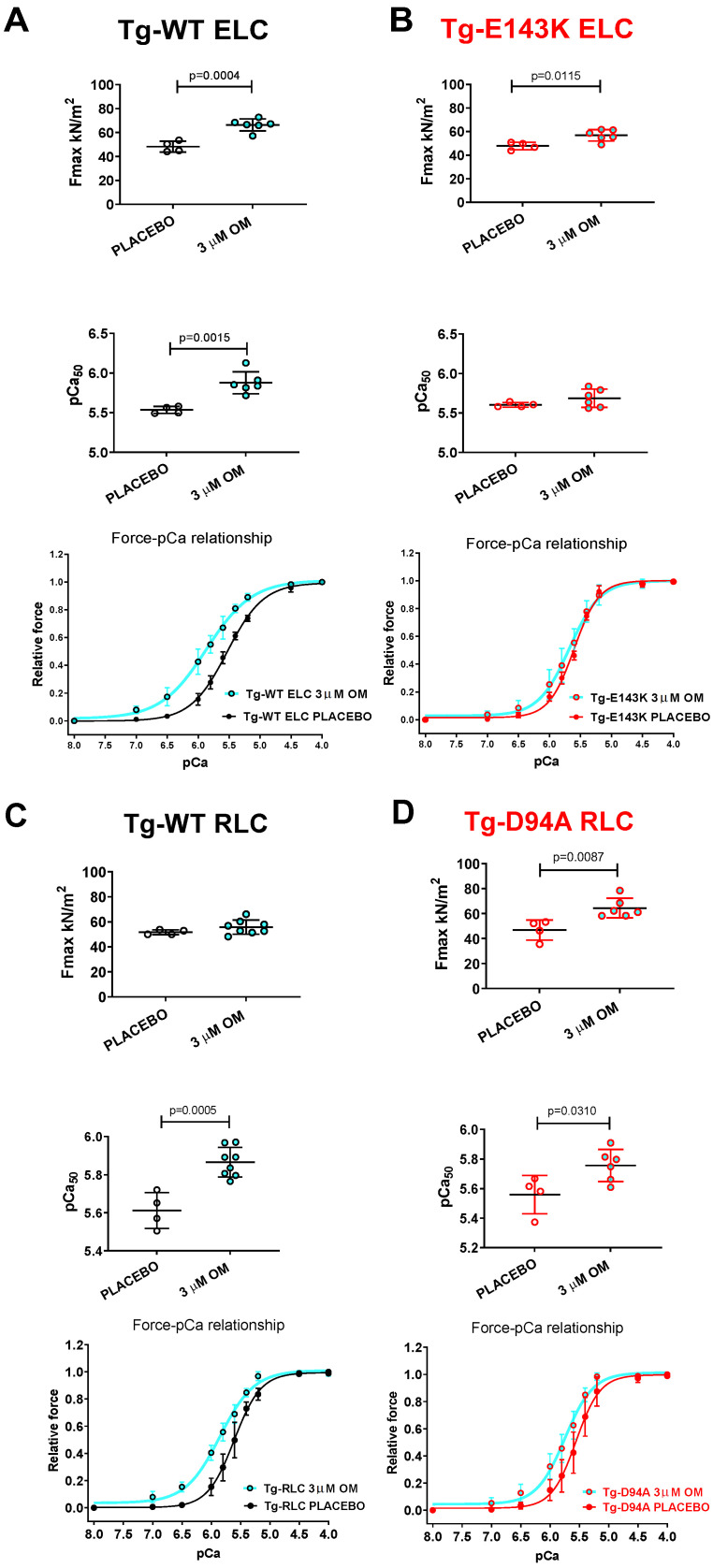
Contractile function in skinned LVPM fibers from Tg-WT ELC (**A**), Tg-E143K ELC (**B**), Tg-WT RLC (**C**), and Tg-D94A RLC (**D**) mice treated with 3 µM OM versus placebo (pCa 8 buffer). Maximal force (top panels), Ca^2+^-sensitivity of force (middle panels), and force–pCa relationships (bottom panels) were measured in fibers treated with 3 µM OM (cyan-filled points) compared with placebo (pCa 8 solution) (clear points). Data are the average ± SD of n = 4–8 fibers (1–2 mice) per group. *p* values were calculated by Student’s *t*-test.

LVPM isolated from all Tg mouse models was subjected to treatment with 3 µM OM, and the results were compared with fibers treated with a placebo (pCa 8 buffer). Exposure of LVPM fibers from Tg-WT ELC and Tg-E143K ELC mice to OM resulted in significantly higher maximal isometric force per cross-section of muscle (in kN/m^2^) (Figure 4A,B, Table 4). Additionally, treatment with OM resulted in a significantly higher calcium sensitivity (larger pCa_50_) of force for Tg-WT ELC, while no change was observed for Tg-E143K ELC fibers (Figure 4A,B, Table 4).

On the other hand, treatment with 3 µM OM of LVPM fibers from Tg-D94A RLC mice resulted in significantly higher maximal pCa 4 force per cross-section of muscle fibers and higher Ca^2+^ sensitivity of force in Tg-WT RLC and Tg-D94A RLC mice compared with placebo-treated fibers (Figure 4C,D, Table 4). 

#### 3.3.2. SRX↔DRX Equilibrium in LVPM Fibers from RCM and DCM Mice 

**Figure 5:**
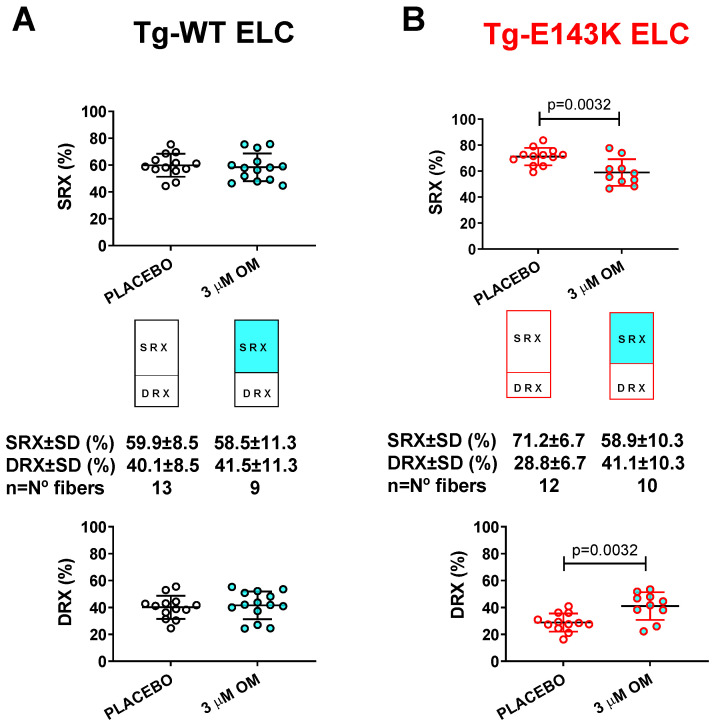

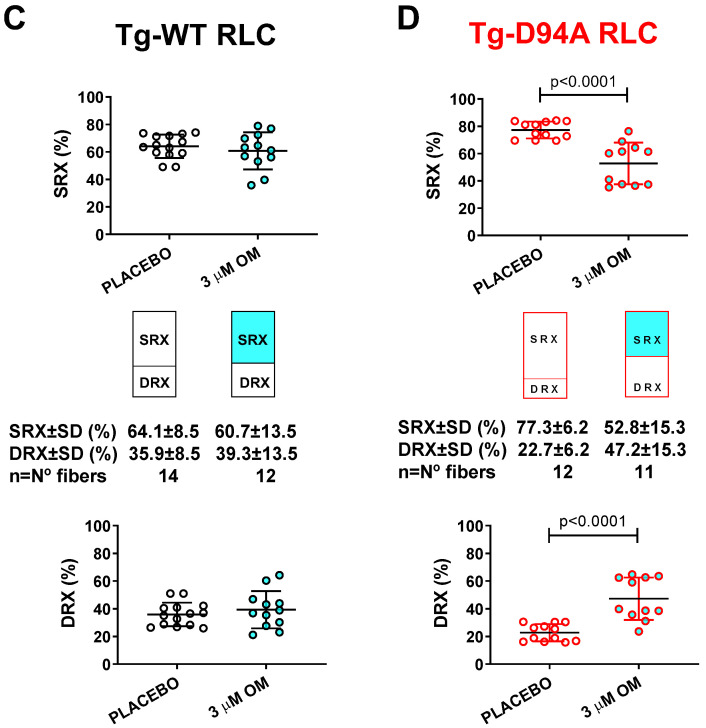
Rescue of SRX↔DRX equilibrium in LVPM from RCM-ELC and DCM-RLC mouse models treated with 3 µM OM. Fibers treated with 3 µM OM (cyan-filled points) were compared with placebo-treated controls (clear points). (**A**) Tg-WT ELC, (**B**) Tg-E143K ELC, (**C**) Tg-WT RLC, and (**D**). Tg-D94A RLC mice. Data are the average ± SD of n = 9–14 fibers of 1–2 mice per group. *p* values were calculated by Student’s *t*-test.

Next, we tested the effect of treatment with 3 µM OM of LVPM fibers from Tg RCM and DCM mouse models and their respective Tg-WT mice and the balance between the SRX and DRX states in OM- versus placebo- (pCa 8 buffer) treated fibers (Figure 5). Despite the significant impact of OM on muscle contraction in transgenic WT-ELC and WT-RLC mice (Figure 4A,C), no effect upon treatment with OM versus placebo was observed in relaxed muscle fibers, which maintained their SRX-to-DRX ratios (Figure 5A,C, Table 5). However, LVPM fibers from RCM-ELC and DCM-RLC mutant mice demonstrated a significantly lower % SRX heads with OM treatment compared with placebo-treated fibers (Figure 5B,D, Table 5). As we showed previously, both models, Tg-E143K ELC and Tg-D94A RLC, favored the energy conservation SRX state, where myosin heads cycled ATP with highly inhibited rates [31,35]. In both models, treatment with 3 µM OM caused a switch from the SRX to the DRX state, where more myosin heads became disordered, relaxed, and readily available for interaction with actin and force production (Table 5). These results indicated a drug-related rescue of the RCM-E143K ELC (Figure 5B) and DCM-D94A RLC (Figure 5D) disease phenotypes.

## 4. Discussion

Myosin RLC phosphorylation is a critical determinant of myosin motor function and heart performance in normal healthy hearts and plays a crucial role in cardiomyopathy. Genetic mutations in contractile proteins, including cardiac myosin RLC and ELC, have been implicated in familial cardiomyopathies, and many of them lower myosin phosphorylation occurring at the N-terminus of myosin RLC (Ser-15) [11,48,49,50]. The pseudo-phosphorylation of myosin RLC by substituting Asp acid for Ser-15 has been widely used in vitro and in vivo as a suitable strategy for normalizing RLC phosphorylation [51]. Our lab has conducted several studies where the pseudo-phosphorylated variant (S15D) of the human ventricular RLC was able to rescue heart function in cardiomyopathy mice [12,22] and in reconstituted LVPM systems [13,21,33]. Still, little is known about the primary mechanisms underlying the beneficial effects of RLC phosphorylation. We hypothesized that one of the mechanisms involves regulating the myosin’s super-relaxed state, which is essential to modulating force production and energy utilization in cardiac sarcomeres [43,52]. Particularly, we hypothesized that the mutation-specific redistribution of myosin energetic states and abnormal SRX↔DRX equilibrium is one of the key mechanisms underlying the pathogenesis of HCM, RCM, or DCM, and successful therapy should target an anomalous SRX/DRX ratio.

We have recently shown the beneficial effect of phosphomimetic S15D-RLC when reconstituted in LVPM fibers from Tg-R58Q RLC and Tg-D166V RLC mice [13,21,33]. In the current report, we aimed to test whether S15D-RLC may rescue RCM-related Tg-E143K ELC and DCM-related Tg-D94A RLC phenotypes when reconstituted in cardiac fibers from mice. We demonstrated that LVPM fibers from Tg-E143K mice decreased the % SRX heads upon reconstitution with the phosphomimetic S15D-RLC compared with WT-RLC reconstitution. Similarly, the abnormal SRX/DRX ratio found in the Tg-D94A RLC model previously [35] returned to normal when the endogenous RLC was replaced by recombinant phosphomimetic S15D-RLC protein. Therefore, the pseudo-phosphorylation of RLCs in the RCM-ELC and DCM-RLC models promoted a shift in the SRX↔DRX equilibrium toward the DRX state, making more heads available for muscle contraction, highlighting the rescue potential of the phosphomimetic S15D-RLC variant. 

The impact of the phosphomimetic S15D-RLC was compared with Omecamtiv Mecarbil, a positive cardiac inotrope, testing its effect on Ca^2+^-activated muscle contraction and the SRX-to-DRX ratio in resting cardiac muscle. In accordance with experiments targeting cardiac myosin contractility [44], the exposure of LVPM fibers from Tg-WT ELC and Tg-E143K ELC mice to OM resulted in significantly higher maximal isometric force per cross-section of muscle and significantly higher calcium sensitivity of force in Tg-WT ELC mice. A similar effect was observed in OM-treated LVPM fibers from Tg-D94A RLC mice, where significantly higher maximal pCa 4 force per cross-section of muscle and higher Ca^2+^ sensitivity of force in Tg-WT RLC and Tg-D94A RLC mice were measured. 

Similarly to the reconstitution of LVPM with S15D-RLC, treatment with 3 µM OM caused a shift from the SRX to DRX state in RCM-E143K ELC and DCM-D94A RLC myocardium. Both models favored the energy conservation state where myosin heads cycle ATP with highly inhibited rates [31,35]. Treatment with the drug produced more myosin heads occupying the DRX state and decreased the number of slowly cycling SRX heads, indicating a drug-related rescue of the RCM-E143K ELC and DCM-D94A RLC phenotypes. 

Our collective results suggest that the number of functionally accessible myosin cross-bridges for their interaction with actin-containing thin filaments is the primary determinant of the power output, and different disease-causing mutations may differently impact the equilibrium between the ON↔OFF states. The clinical phenotypes associated with cardiomyopathies discussed in this report are expected to be improved following S15D-RLC or OM treatment. Biochemical destabilization of the SRX with both therapeutic agents is anticipated to enhance myosin activity via increasing the rate of ATP turnover and accelerating Pi release, which is the rate-limiting step in the actin–myosin ATPase cycle. Destabilizing the SRX state may lead to an increased pool of myosin heads that bind to actin in a force-producing state.

## Figures and Tables

**Table 1:** Maximal tension and force–pCa relationship in LVPM fibers from Tg-WT ELC and Tg-E143K ELC mice reconstituted with recombinant WT and S15D RLC proteins.

Force Parameter/ Recombinant RLC-Reconstituted LVPM	LVPM from Tg-WT ELC Mice		LVPM from Tg-E143K Mice	
	WT	S15D	WT	S15D
N° fibers	8	7	9	10
Fmax (kN/m^2^) ± SD	21.56 ± 4.64	34.25 ± 9.66 **	23.56 ± 4.42	28.58 ± 1.95
pCa_50_ ± SD	5.32 ± 0.1	5.41 ± 0.06 *	5.51 ± 0.09	5.51 ± 0.09
n_H_ ± SD	2.03 ± 0.74	1.87 ± 0.41	2.2 ± 0.61	1.73 ± 0.24 *

Values are means ± SD for n = N° fibers. Significance was calculated by Student’s *t*-test with * *p* < 0.05, ** *p* < 0.01 for S15D-RLC versus WT-RLC reconstituted LVPM fibers.

**Table 2:** The SRX state of myosin assessed in LVPM fibers from Tg-WT ELC and Tg-E143K ELC mice reconstituted with recombinant WT and S15D RLC proteins.

SRX Parameter/Recombinant RLC-Reconstituted LVPM	LVPM from Tg-WT ELC Mice			LVPM from Tg-E143K ELC Mice		
	Native	WT	S15D	Native	WT	S15D
N° fibers	14	6	7	13	11	10
DRX (%) ± SD	38.9 ± 8.2	35.1 ± 14.2	37.2 ± 18.8	29.4 ± 3.7 ^##^	32.3 ± 11.9	44 ± 12.8 *
SRX (%) ± SD	61.1 ± 8.2	64.9 ± 14.2	62.8 ± 18.8	70.6 ± 3.7 ^##^	67.8 ± 11.9	56 ± 12.8 *
T1 (s) ± SD	4.4 ± 2.4	6.6 ± 4.9	5.3 ± 2.5	4.2 ± 2.5	4.8 ± 3.6	7 ± 5.2
T2 (s) ± SD	129.8 ± 67	125.3 ± 81.3	173.4 ± 167.7	126.5 ± 81.2	92.2 ± 64	136.4 ± 116.5

Values are means ± SD for n = N° fibers. Significance was calculated by one-way ANOVA with Tukey’s multiple-comparison test with * *p* < 0.05 for LVPM fibers from Tg-E143K mice reconstituted with S15D-RLC versus WT-RLC. ^##^ *p* < 0.01 for native Tg-E143K ELC versus Tg-WT ELC mice.

**Table 3:** The SRX state of myosin assessed in LVPM fibers from Tg-D94A RLC mice reconstituted with recombinant WT, S15D, and D94A RLC proteins.

SRX Parameter/Recombinant RLC-Reconstituted LVPM	LVPM from Tg-D94A RLC Mice			
	Native	WT	S15D	D94A
No. fibers	13	13	8	12
DRX ± SD	26.3 ± 7.3	44.9 ± 10.2 ****^,^^	46.7 ± 13.2 ****^,^^	33.6 ± 6.4
SRX ± SD	73.7 ± 7.3	55.1 ± 10.2 ****^,^^	53.3 ± 13.2 ****^,^^	66.4 ± 6.4
T1 ± SD	6.5 ± 3.3	4.9 ± 2.8	5.7 ± 5.1	2.7 ± 1.7 *
T2 ± SD	161.8 ± 149	136.9 ± 103.1	117.7 ± 130.8	67.4 ± 77.3

Values are means ± SD of n = N° fibers. Significance was calculated by one-way ANOVA with Tukey’s multiple comparison test; **** *p* < 0.0001, * *p* < 0.05 for fibers reconstituted with recombinant RLC proteins versus native; ^ *p* < 0.05 for WT or S15D mutant versus D94A-RLC-reconstituted.

**Table 4:** Maximal tension (pCa 4) and force–pCa relationship in LVPM fibers from Tg-WT ELC, Tg-E143K ELC, Tg-WT RLC, and Tg-D94A RLC mice treated with Omecamtiv Mecarbil versus placebo.

Force Parameter/ OM- vs. Placebo-Treated Fibers	LVPM from Tg-WT ELC Mice		LVPM from Tg-E143K ELC Mice		LVPM from Tg-WT RLC Mice		LVPM from Tg-D94A RLC Mice	
	OM	Placebo	OM	Placebo	OM	Placebo	OM	Placebo
No. fibers	6	4	6	4	8	4	6	4
Fmax (kN/m^2^) ± SD	66.4 ± 5.1 ***	48.3 ± 4.5	56.8 ± 4.8 *	47.8 ± 3.2	55.8 ± 5.7	51.7 ± 2	64.5 ± 7.8 **	46.9 ± 8.1
pCa50 ± SD	5.9 ± 0.1 **	5.5 ± 0.04	5.7 ± 0.1	5.6 ± 0.03	5.9 ± 0.1 ***	5.6 ± 0.1	5.8 ± 0.1 *	5.6 ± 0.1
n_H_ ± SD	1.2 ± 0.1 *	1.5 ± 0.2	1.8 ± 0.2 *	2.2 ± 0.1	1.5 ± 0.2 **	2 ± 0.2	1.9 ± 0.3 *	2.2 ± 0.2

Values are means ± SD of n = N° fibers. Significance was calculated by Student’s *t*-test with * *p* < 0.05, ** *p* < 0.01, and *** *p* < 0.001 for OM- versus placebo-treated fibers.

**Table 5:** The SRX study in LVPM from Tg-WT ELC, Tg-E143K ELC, Tg-WT RLC, and Tg-D94A RLC mice treated with OM versus placebo.

SRX Parameter/ OM- vs. Placebo-Treated Fibers	LVPM from Tg-WT ELC Mice		LVPM from Tg-E143K ELC Mice		LVPM from Tg-WT RLC Mice		LVPM from Tg-D94A RLC Mice	
	OM	Placebo	OM	Placebo	OM	Placebo	OM	Placebo
No. fibers	14	13	10	12	12	14	11	12
DRX ± SD	41.6 ± 10.4	40.1 ± 8.5	41.1 ± 10.3 **	28.8 ± 6.7	39.3 ± 13.4	35.9 ± 8.5	47.2 ± 15.3 ****	22.7 ± 6.2
SRX ± SD	58.4 ± 10.4	59.9 ± 8.5	58.9 ± 10.3 **	71.2 ± 6.7	60.7 ± 13.4	64.1 ± 8.5	52.8 ± 15.3 ****	77.3 ± 6.2
T1 ± SD	3.7 ± 3.4	3.2 ± 1.8	3.7 ± 2.4	4.2 ± 2.7	4.2 ± 3	5.6 ± 3.8	3.7 ± 1.9 *	6.9 ± 4.1
T2 ± SD	101.4 ± 152.5	69 ± 38.5	89.3 ± 78.9	112.9 ± 94.3	123.8 ± 86.8	119.6 ± 122.4	83.1 ± 65.6 *	230.6 ± 213.8

Values are means ± SD of n = N° fibers. Significance was calculated by Student’s *t*-test with * *p* < 0.05, ** *p* < 0.01, **** *p* < 0.0001 for OM- versus placebo-treated LVPM.

## Data Availability

Data are contained within the article or Supplementary Material.

## Supplementary Materials

The following supporting information can be downloaded at: https://www.mdpi.com/article/10.3390/life13071463/s1, Figure S1: Representative SDS-PAGE of RLC-depleted and mutant RLC-reconstituted LVPM preparations from Tg-WT ELC, Tg-E143K ELC, and Tg-D94A RLC mice.
