# Supplementary material for: Phosphorylation Mimetic of Myosin Regulatory Light Chain Mitigates Cardiomyopathy-Induced Myofilament Impairment in Mouse Models of RCM and DCM
Source: Life (Basel). 2023 Jun 28;13(7):1463. doi: 10.3390/life13071463 (PMC10381296; doi:10.3390/life13071463)
Supplement: Supplementary file 1 [file life-13-01463-s001.zip › life-2459288-supplementary.pdf]

Supplementary Materials

Figure S1

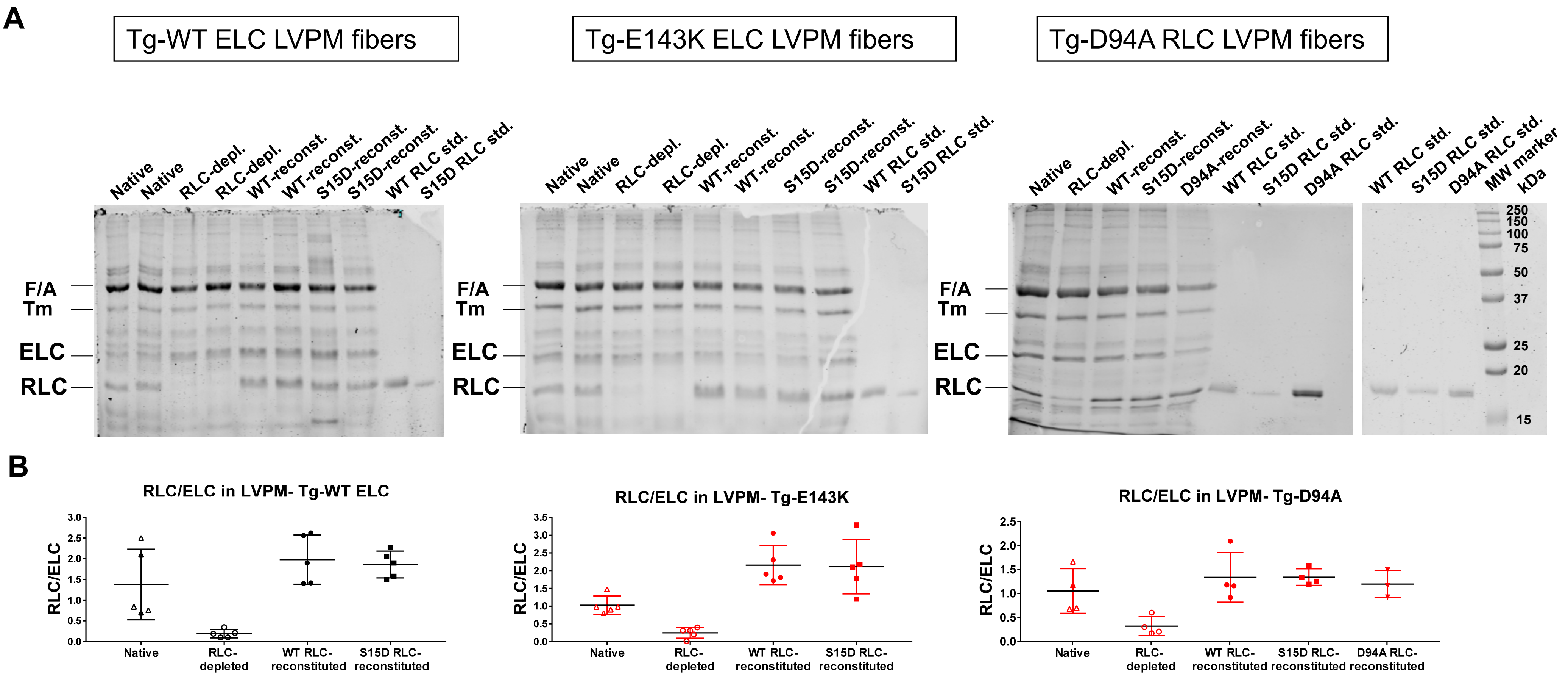

SDS-PAGE images of LVPM fibers from Tg-WT ELC, Tg-E143K ELC, and Tg-D94A RLC mice were RLC-depleted and mutant-reconstituted. Depletion and reconstitution experiments in Tg-WT ELC, Tg-E143K ELC, and Tg-D94A RLC LVPM fibers are shown in (A), while quantification is presented in (B). LVPM fibers were CDTA/Triton-depleted of endogenous RLC and reconstituted with recombinant WT-RLC, S15D-RLC, and D94A-RLC proteins. On average, 3-5 LVPM fibers per group were subjected to depletion/reconstitution. The band intensity of ELC and RLC proteins were assessed, and the RLC/ELC ±SD ratios were calculated: 1.38±0.85 for Tg-ELC native, 1.06±0.22 for Tg-E143K ELC native, and 1.05±0.47 for Tg-D94A RLC native. RLC/ELC ±SD ratios for depleted fibers were 0.20±0.10 for Tg-ELC, 0.24±0.15% for Tg-E143K, and 0.32 ±0.20 for Tg-D94A. RLC/ELC ±SD ratios for reconstituted fibers were 1.92±0.45 for Tg-ELC, 2.13±0.62 for Tg-E143K, and 1.30±0.33 % for Tg-D94A. Abbreviations: F/A, F-actin; Tm, Tropomyosin; ELC, myosin essential light chain (*MYL3* gene); and RLC, myosin regulatory light chain (*MYL2* gene).
